# Impacts of Single and Multiple Co‐Existing Invasive Species on Subtropical Native Ant Communities

**DOI:** 10.1002/ece3.72095

**Published:** 2025-09-01

**Authors:** Jiaxin Hu, Taylor A. Bogar, Matthew T. Hamer, Benoit Guénard

**Affiliations:** ^1^ School of Biological Sciences The University of Hong Kong Hong Kong SAR China; ^2^ Science Unit Lingnan University Hong Kong SAR China

**Keywords:** biological invasion, co‐invasion, functional diversity, functional trait, insect, invasion intensity

## Abstract

Biological invasions pose a significant threat to ecosystem stability by altering the taxonomic and functional diversity of native communities. It is still uncertain, however, whether multiple invasive species have varying effects on native communities, or whether their interactions in a co‐invasion scenario are antagonistic or facilitative. To address this gap, this study investigated 24 sampling sites in Hong Kong, encompassing single invasion, co‐invasion, and non‐invaded control scenarios across the dry and wet seasons. We systematically explored how the functional traits and invasion intensity of four invasive ant species (*Anoplolepis gracilipes, Paratrechina longicornis, Pheidole megacephala, and Solenopsis invicta*) shape the structure and function of native ant communities. In addition, we evaluated the ecological effects of co‐invasion scenarios to determine how interactions between invaders affect communities. The results revealed that, for some invasive species, increased invasion intensity significantly reduced local species diversity and resulted in selective replacement and systematic loss of local species. The invasion intensity of three invasive species had significantly negative effects on functional diversity indices (e.g., RaoQ and FDiv), while in areas invaded by *P. megacephala* , the near absence of native species (present in ~4% of the pitfall traps) prevented analyses. Comparisons across non‐invaded, single invasion, and co‐invasion scenarios revealed distinct patterns: single invasions caused pronounced reductions in both functional and taxonomic diversity, whereas co‐invasion scenarios exhibited more limited changes. Further analyses demonstrated that under specific circumstances, antagonistic interactions between co‐invasive species could mitigate the negative effects on α‐diversity and preserve ecosystem functions through functional substitution. Nevertheless, this dynamic equilibrium is fragile and unsustainable, underscoring the need to prioritize preventive and control strategies in invasive species management to safeguard ecosystem resilience.

## Graphical abstract

This study examines the impact of invasive ant species on native ant communities in Hong Kong, highlighting that single‐species invasions often lead to significant declines in biodiversity. In certain cases, co‐invasions may mitigate these effects; however, such equilibrium is fragile and unsustainable. Consequently, the findings underscore the necessity of prioritizing preventive and management strategies to safeguard ecosystem stability.

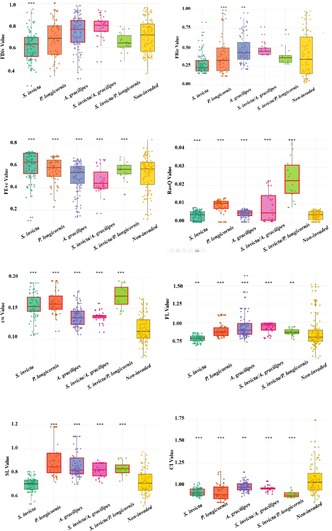

## 1 Introduction

Biological invasions are recognized as an important driver affecting ecosystem stability and have become a central topic in ecological and conservation research (Ehrenfeld 2010; Strayer 2012; Simberloff et al. 2013; Moodley et al. 2022; Vantarová et al. 2023; Carneiro et al. 2024; Haubrock et al. 2024). Non‐native species can alter resource allocation patterns and ecological processes of invaded communities through mechanisms such as competitive exclusion, resulting in increased pressure on the survival of native species (Kimbro et al. 2009; Walsh et al. 2016; Ficetola et al. 2024; Devenish et al. 2025). These changes not only inhibit the survival of native species in the community, but also reshape species composition and functional structure, further weakening the stability and diversity of the species community (Mooney and Cleland 2001; Wang et al. 2021).

Non‐native species have significant impacts on the taxonomic and functional diversity of native species, affecting both species richness, abundance, and trait distribution (Davies 2011; Laverty et al. 2017). Typically, invasive species show higher resource use efficiencies, limiting the niche space of native species and promoting homogenization of community functions, further increasing the risk of extinction of native species (Blackburn et al. 2019; Tordoni et al. 2019; Wong et al. 2020). This process ultimately leads to a homogenization of functional characteristics, causing communities to lose their resilience and stability and posing a threat to the long‐term survival of native species (Muthukrishnan and Larkin 2020; Wong et al. 2020). Notably, the negative impacts of invasive species on communities are exacerbated as the density of invasion increases. For instance, the negative impacts of the predatory invasive fish species *Pterois volitans* on native species biomass and abundance in the Caribbean Sea increased nonlinearly with increasing invasion density (number of invasive individuals per unit area) (Benkwitt 2015).

The impacts of invasive species on native communities, however, differ substantially in function of the invader identity and the community being invaded. For example, some invasive plants (woody invaders) significantly weaken the structure and function of native arthropod communities, while others (herbaceous invaders) present relatively limited effects (Van Hengstum et al. 2014). This discrepancy may stem from differences in the ecological niche characteristics of invasive plants, leading to significant differences in their mechanisms of action on arthropod communities. Similarly, these differences are also observed in the alteration of structural composition and functional attributes of invaded communities. According to Kaushik et al. (2022), the performance of invasive species in dynamic ecosystems is largely determined by differences in their functional traits. For instance, in windy sub‐Antarctic environments, invasive plant species tend to exhibit lower plant height compared to native species, a functional trait conferring them a competitive advantage (Mathakutha et al. 2019). The differences in functional traits not only alter the dynamics of competition between invasive and native species but may also further affect the functional diversity of the community by promoting or suppressing functional traits of native species. Further studies on invaded communities suggested that native species may coexist with invasive species if they have high competitiveness traits in resource acquisition similar to those of invasive species. Therefore, the mean functional traits of the resident species in the invaded community gradually move closer to the mean functional traits of the invasive species. This change brings the community‐weighted mean closer to the invasive species‐weighted mean (Gallien et al. 2015; Fried et al. 2019).

Globally, biological invasions remain very dynamic, with well‐identified, as well as new species constantly invading new regions due to the global trade between nations (Seebens et al. 2018). Ultimately, this leads native communities to be invaded by multiple invasive species. Although extensive research has explored the impacts of single invasive species on local biodiversity (Vilà et al. 2011; Pyšek et al. 2012), studies addressing the combined effects of co‐invasion on local communities are more limited (Kuebbing et al. 2013; Chang et al. 2018; Móréh et al. 2024). Co‐invasions can disrupt local communities in a more destructive way than a single invasive species through complex mechanisms, leading to unbalanced resource allocation, altered interactions between species, and significant degradation of community functions (LeBrun et al. 2013; Jackson 2015; Fryxell et al. 2016; Lenda et al. 2023; Lone et al. 2024). These synergistic effects can include accelerated rates of habitat alteration, increased competition for resources, and greater overall native species' survival stress. For instance, Fryxell et al. (2016) and Lone et al. (2024) documented synergistic effects of co‐invasions where ecological disruption exceeded or equals the sum of single species impacts. However, Lenda et al. (2023) and Jackson (2015) identified cases of antagonistic interactions that moderated negative impacts on native communities. The combined effects of co‐invaders may weaken the adaptive capacity and viability of native species within communities, ultimately leading to more pronounced declines in community diversity and functional structure (Fryxell et al. 2016).

Alternatively, the presence of multiple invasive species can result in antagonistic and regulatory relationships, which as a result mitigate some of the individual invasive species detrimental effects on local communities. For example, invasive species may inhibit the spread or establishment of other invasive species through competitive interactions, thereby reducing their overall negative impacts (Li et al. 2024). These competitive dynamics then create a form of biological control, where one invasive species limits the success of another, potentially providing native species with a reprieve and an opportunity to recover. For example, Wang et al. (2020) showed a significant antagonistic effect of plant co‐invasion between *Erigeron annuus* and *Solidago canadensis* , increasing the level of Mason's α functional diversity in the community. Similarly, the invasion of *Nylanderia fulva* in the southern United States can lead to a decrease in populations of the Red Imported Fire Ant, *Solenopsis invicta* (LeBrun et al. 2013, 2014). This suggests that antagonistic mechanisms between invasive species not only alleviate the pressure faced by native species communities, or a part of them, but may also have aided community recovery to some extent, enhancing species abundance and diversity.

Hong Kong stands as a major trade hub globally, which has rendered it a hotspot for biological invasions (Wong et al. 2022). A prominent example is the invasion of ants, with four major invasive species— *Anoplolepis gracilipes* , *Paratrechina longicornis* , *Pheidole megacephala* , and *S. invicta* —currently recorded in the region. These invasive ant species are known to disrupt native ant populations in various regions and lead to cascading effects on the broader ecological community (Drescher et al. 2011; Roeder et al. 2021; Tercel et al. 2023). However, much of the current research has focused on the effects of a single invasive species, principally *S. invicta* and its impacts on the functional structure and composition of local ant communities (Wong et al. 2020, 2022). In this study, we aimed at gaining a deeper understanding of the impacts of several widespread invasive ants on local communities, considering both single‐species and multi‐species invasion scenarios across the site and individual pitfall trap levels. We posed the following hypotheses: (1) The taxonomic and functional diversity of invaded communities is expected to vary as a function of the identity of the invasive ant species. Some invasive species may show limited impacts, while others may cause profound changes in the taxonomic and functional diversity of local communities. (2) The impact of invasive species on native communities may be mediated by the interaction between their functional traits and invasion intensity. For instance, specialized functional traits such as long legs, long scape (efficient in acquiring food), or well‐developed mandibles (cutting or carrying food efficiently) may confer a significant competitive advantage or higher resource acquisition capacity to invasive species (Pearce‐Duvet et al. 2011; Richter et al. 2019), thereby exacerbating the effects on community function and structure. (3) There may be potential inhibitory relationships (antagonism) between co‐invading invasive species, thereby reducing the negative impact on native communities. However, while in some cases antagonism significantly mitigates the negative effects of co‐invasion, in most cases it should only partially offset these effects without fully eliminating them.

## 2 Materials and Methods

This study was carried out in Hong Kong from 2022 to 2024 and included 24 sampling sites distributed across various locations, including forest edges adjacent to villages or agricultural lands. The sites included four sites invaded by *Solenopsis invicta* , four sites invaded by *Pheidole megacephala* , four sites invaded by *Anoplolepis gracilipes* , and four sites invaded by *Paratrechina longicornis* . Additionally, there were four sites with no invasive species and four sites containing two invasive species (three sites inhabited by *Solenopsis invicta* and *Anoplolepis gracilipes* , one site with *Solenopsis invicta* and *Paratrechina longicornis* ). In total, 24 sites were sampled for an entire year (2 sampling seasons), while an additional 9 and 3 sites were resampled for a second year during the wet and dry seasons, respectively (see Table S3 in Appendix S1). To reduce potential overlap in species foraging ranges and colony interactions, particularly in polydomous or supercolony‐forming invasive ants, the distance between neighboring sites was maintained at a minimum of 50 m.

Systematic sampling was conducted at all sites during both the wet and dry seasons to facilitate a comparative analysis of the results under different seasonal and environmental conditions. During each sampling session, either during the wet (hot and humid) and dry (cooler and dry) seasons, we deployed 12 pitfall traps for a total of 24 pitfall traps per site Pitfall traps were spaced 5 m apart, with this interval 2.5 times larger than the average foraging distance of most species in the region (Eguchi et al. 2004). The traps were placed flush with the ground and contained a solution of soapy water. After 48 h, we collected the traps and sorted, identified, and counted the captured ants. A total of 720 traps were deployed throughout the study period. However, only 646 traps were used for analyses due to the destruction of some traps by wildlife (e.g., wild boar; *N* = 41) or the complete absence of ants during the cooler temperatures of the dry season (*N* = 76). All specimens were sorted into morphospecies and subsequently identified to species using taxonomic keys.

### 2.1 Assembling the Individual‐Level Trait Dataset

In this study, we aimed to obtain functional diversity values that include intraspecific trait variation, specifically variations arising from worker polymorphism. To achieve this, we assembled an individual‐level trait dataset comprising data on eight morphological traits likely to influence ant physiology and behavior, hypothesizing that these traits would impact ant performance and adaptability (see details in Table 1). Using specimens collected from pitfall traps, we captured high‐resolution images and conducted trait measurements with a stereomicroscope equipped with Leica Application Suite V4 software. A total of 1283 individual ants were measured, and for each monomorphic species, at least 5 specimens were included to ensure a representative sample. For dimorphic or polymorphic species, we measured 4 to 10 minors as well as 1 to 2 majors for each species. Before conducting all statistical analyses, we performed size correction on the following traits (pronotum width, eye width, mandible length, scape length and femur length) by dividing their values by body size (measured as Weber's length; see Table S2 in Appendix S1). The cephalic index was measured by dividing head width by head length (Kikuchi et al. 2008). With the updated trait dataset containing size‐corrected values for six traits, we applied logarithmic transformations to mitigate the influence of extreme values and standardized the trait values to achieve a mean of zero and unit variance.

**TABLE 1:** Effects of single and combined invasive ant species on native species abundance (NSA), native species richness (NSR), and α diversity under wet and dry conditions. In the α diversity, bold text indicates statistically significant differences between invaded and uninvaded pitfall traps. Additionally, the asterisk indicates that the impact of invasion intensity on the value is statistically significant (***p < 0.001, **p < 0.01).

	*S. invicta*	*A. gracilipes*	*P. longicornis*	*P. megacephala*	*S. invicta/A. gracilipes*	*S. invicta/P. longicornis*
NSA (wet)	−0.23**	−0.24	0.011	−1.65***	−0.26*	0.25
NSR (wet)	−0.29*	−0.01	0.113	−1.83***	−0.43*	0.32
NSA (Dry)	−0.06	−0.07	0.006	−2.34***	−0.03	0.12
NSR (Dry)	−0.07	−0.02	0.004	−2.67***	−0.006	0.03
α diversity	−0.88***	−0.85***	−1.12***	**−3.53**	2.66***	2.42**

Principal Component Analysis (PCA) was employed to capture the major axes of variation within the multidimensional trait space, thereby reducing the dimensionality for calculating species‐level functional richness and divergence. The PCA was conducted using the mean trait values of each species, and the PCA component values were subsequently predicted for all individuals in the dataset. We retained the first two principal components that met the broken‐stick criterion (Peres‐Neto et al. 2003). The first principal component explained 33.6% of the total variation found in traits and was strongly positively correlated with scape length and femur length and negatively correlated with Weber's length. The second principal component explained 25.8% of the variation and was positively correlated with pronotum width and mandible length and negatively correlated with Weber's length. We predicted the values of these two components for every individual in the trait dataset and used these new ‘traits’ to calculate functional diversity indices.

### 2.2 Functional Diversity From Species to Communities

We calculated a community‐weighted mean (CWM) for each corrected functional trait (Lavorel et al. 2008). The CWM was obtained by weighting the average trait value for each species, where the weights were determined by species occurrence frequencies at each site (number of traps with a particular species/total number of traps within each site). The CWM provides a measure of the community's dominant trait values, reflecting the functional traits that are dominant throughout the community (Lavorel et al. 2008; Ricotta and Moretti 2011). This approach integrates the contributions of individual species and ensures a more accurate representation of the overall functional characteristics of the community.

All functional diversity indices were calculated using a trait probability density framework that integrates intraspecific variation, trait dimensionality, species abundance, and trait probability distributions. This comprehensive approach ensures that the diversity measures accurately reflect the complexity of biological traits within and among species. We calculated the means and standard deviations of the ant species. To minimize errors, we utilized the ‘TPDs’ function from the ‘*TPD*’ package in *R*. This function stands out for its ability to compute intraspecific probabilities by using the multivariate normal distribution for each species or population. This method offers a significant advantage over the more commonly used kernel density estimation, which can be less accurate when dealing with unevenly distributed data (Carmona et al. 2019). Subsequently, we calculated the TPDcom (local community levels) using the ‘TPDc’ function. Functional diversity indices for each community's TPDcom were calculated using five different metrics (see Table S4 in Appendix S1) including functional richness, functional evenness, functional redundancy, functional divergence, and Rao's quadratic entropy (Carmona et al. 2016, 2019). Functional Richness (Fric) represents the total volume occupied by the community in the functional space. Functional Evenness (FEve) assesses whether the distribution of the community in the functional space is even. Functional Redundancy (FRed) indicates the extent to which trait values are represented by multiple species within the community. Functional Divergence (FDiv) measures the degree of dispersion of functional traits within the community. Rao's Quadratic Entropy (RaoQ) is a diversity index that comprehensively considers species richness and functional trait differences. It measures the overall degree of functional trait differences among species within the community, reflecting the diversity of functional traits among species.

### 2.3 Impact of Co‐Invasion Effects

In assessing the impacts of co‐invasion of two invasive species on native communities, it is important to synthesize the impacts associated with the invasion of a single species as well as the effects of the co‐invasion interactions. Specifically, this means that not only is it necessary to quantify the impacts of each invasive species on native species when it is present independently, but also to analyze the synergistic effects of their interactions (co‐invasion interactions). Such synergistic effects may exacerbate or mitigate the pressure of invasive species on native communities. Thus, for the co‐invasion, the effects of the two species on the native species are as follows:

(1) $$R=\beta_{1}\times X_{1}+\beta_{2}\times X_{2}+\gamma_{12}\times X_{1}\times X_{2}+b$$

The *R* represents the effect on the community following the invasive species. Here, $X_{1}$ and $X_{2}$ denote the invasion intensity of invasive species 1 and 2, respectively, with values ranging from 0 to 1. Coefficients $\beta_{1}$ and $\beta_{2}$ indicate the linear effects of each invasive species on the community, while the coefficient $\gamma_{12}$ captures the synergistic effects (or antagonistic) of the interaction between the two invasive species on the community. In this equation, b represents the intercept, which indicates the baseline level of the dependent variable when all predictor variables are equal to zero.

When $\gamma_{12}\neq 0$, it indicates the presence of either antagonistic interactions ($\gamma_{12}>0$) or synergistic interactions ($\gamma_{12}<0$). In cases of synergistic interactions, the two invasive species mutually facilitate each other, resulting in a co‐invasion impact that exceeds the combined impact of the two single invasions, thereby intensifying the disruption to the native community.

When $\gamma_{12}>0$ and $\left|\gamma_{12}\right|>\left|\beta_{1}\right|\;\text{or}\;\left|\beta_{2}\right|$, it can be assumed that the invasion intensity of species 1 is a multiple of the invasion intensity of species 2, denoted as $X_{1}=a\times X_{2}$. Thus, the formula is:

(2) $$R=\beta_{1}\times a\times X_{2}+\beta_{2}\times X_{2}+\gamma_{12}\times a\times {X_{2}}^{2}$$

Hence, when

(3) $$\gamma_{12}\times a\times {X_{2}}^{2}>-\left(\beta_{1}\times a\times X_{2}+\beta_{2}\times X_{2}\right)$$

the antagonistic effect is greater than the combined impact of two single invasions. Solving this inequality yields:

(4) $$X_{2}>\frac{-\left(\beta_{1}\times a+\beta_{2}\right)}{\gamma_{12}\times a}$$

Since $X_{2}$ ranges between 0 and 1, the inequality becomes:

(5) $$\frac{-\left(\beta_{1}\times a+\beta_{2}\right)}{\gamma_{12}\times a}\leq 1$$

Thus, we obtain:

(6) $$a\geq \left|\frac{-\beta_{2}}{\gamma_{12}+\beta_{1}}\right|$$

Further, since $X_{1}=aX_{2}\leq 1$ and $X_{2}\leq 1$, we obtain:

(7) $$X_{2}\leq \frac{1}{a}$$

Substituting Inequality (Equation 7) into Inequality (Equation 4), we can obtain:

(8) $$a<\frac{\gamma_{12}+\beta_{2}}{-\beta_{1}}$$

Therefore, when $|\frac{\beta_{2}}{\gamma_{12}-\beta_{1}}|<\frac{X_{1}}{X_{2}}<|\frac{\gamma_{12}+\beta_{2}}{-\beta_{1}}|$, the antagonistic interaction between the two invasive species leads to a total impact smaller than combined impact of the two single invasions, thereby mitigating the effects of invasion on the native community and potentially promoting its biodiversity.

### 2.4 Statistical Analysis

#### 2.4.1 Stratified Analysis of Invasive Species Impact

We conducted a comprehensive study of all collected pitfall traps.

For sites invaded by invasive species, we observed that some pitfall traps failed to capture any invasive species, indicating that the invasive species have not fully occupied these sites. Consequently, we employed a stratified analysis approach in our analysis, specifically including both the pitfall trap and the sample site levels. This two‐tiered method enhances the accuracy and reliability of our research findings, thus providing robust support for the conservation of local species diversity and the maintenance of communities' functions.

#### 2.4.2 Pitfall Trap Level

We conducted a detailed count of the individuals of both invasive and native species within each pitfall trap (species abundance). Additionally, we recorded the number of native species in each pitfall trap (species richness).

##### 2.4.2.1 Impact of Invasive Species Abundance on Native Species Richness and Abundance

In this study, we conducted detailed species surveys for all pitfall traps across the sample sites. Specifically, we recorded the individual counts of invasive species (invasive species abundance), the number of native species (native species richness), and the total count of native species (native species abundance) within each pitfall trap (dry and wet season). Based on these data, we further categorized the pitfall traps across various invasion sites and non‐invaded sites into three categories: (1) Pitfall traps containing single invasive species; (2) Pitfall traps containing multiple invasive species; (3) Pitfall traps with no invasive species detected. This classification allows us to analyze and compare the impact of invasive species on the richness and abundance of native species across different conditions, providing deeper insights into the ecological effects of biological invasions. In addition, we further compared the differences in species diversity and functional diversity between pitfall traps containing invasive species and pitfall traps without invasive species in the invasion sample sites (see Table S1 in Appendix S1).

In order to evaluate the impact of single invasive species on the native species abundance and richness in pitfall trap data across different seasons, we employed Zero‐Inflated Negative Binomial (ZINB) regression models and constructed separate models for each invasive species recorded. To explore the impact of multiple invasive species on native species counts, we conducted statistical analyses using pitfall trap data from all invaded sites, also employing ZINB regression models. This model effectively handles the zero‐inflation present in the data while considering the independent effects and potential interactions of multiple invasive species on the pitfall traps to assess their impact on native species counts.

The ZINB model addresses the high prevalence of zero observations in the dataset while incorporating mixed effects to account for spatial autocorrelation at multiple levels (Minami et al. 2007). Specifically, it considers autocorrelation among pitfall traps within the same study site, as well as temporal autocorrelation among traps deployed in the same site across different years. During data preprocessing, we applied a logarithmic transformation (log (*x* + 1)) to all species count data to ensure consistency in data variance. This standardization process effectively reduces data skewness and kurtosis, ensuring the reliability of the model fitting results.

##### 2.4.2.2 Impact of Invasion Intensity on Taxonomic Diversity

In this study, we calculated the individual counts of invasive species across different invaded sites. To achieve data standardization, we divided the abundance of invasive species by the total abundance of ants in each pitfall trap, obtaining a standardized value representing the invasion intensity. Next, we employed the Shannon Index to quantify α diversity at each pitfall trap and used the Mann–Whitney U Test to examine differences between invasion and non‐invasion scenarios. We then utilized Generalized Linear Mixed‐Effects Models (GLMMs) to analyze the impact of invasion intensity on α diversity, incorporating sampling site and year as random effects to account for spatial and temporal autocorrelation among pitfall traps. This analysis included evaluating the effects of various single species invasion intensities on the α diversity of invaded pitfall traps, as well as the impact of multiple species invasions on α diversity. Since both the diversity index and invasion intensity fall within the range of [0, 1], the application of GLMMs offers significant advantages in data processing. We further calculated beta diversity between pitfall traps using the Bray–Curtis dissimilarity matrix and employed distance‐based redundancy analysis (9999 permutations) to evaluate the relationships between different invasion intensities and community composition, turnover, and nestedness. By including site as a factor in the distance‐based redundancy analysis, we effectively controlled for the influence of spatial heterogeneity on the results, thereby enhancing the reliability of our conclusions.

##### 2.4.2.3 Impact of Invasion Intensity on Functional Diversity

We also quantified the invasion intensity of invasive species within each pitfall trap across different invaded sites. The counts of all species in the traps were log‐transformed (log(*x* + 1)). We used the TPDcom method to quantify functional diversity indices in different pitfall traps, including Fric, FEve, FRed, FDiv, and RaoQ. Additionally, we employed Generalized Linear Mixed‐Effects Models (GLMMs) to analyze the impact of varying invasion intensities on functional diversity indices and CWM. Since both functional diversity indices and invasion intensity fall within the [0, 1] range, the application of GLMMs is particularly advantageous in handling zero values and considering random effects from sampling sites, thereby enhancing the adaptability and accuracy of the models. We also utilized the Mann–Whitney U Test to examine differences in CWM and functional diversity indices among different invasion scenarios (single species invasion, multiple species invasion, and no invasion).

#### 2.4.3 Site Level

We conducted a detailed analysis of species occurrence frequency within each site. We calculated the proportion of each species captured in pitfall traps (~24 pitfall traps in total) at each site to determine the relative frequency of species within each site.

We employed Mann–Whitney U Tests to evaluate whether there were significant differences in taxonomic diversity, functional diversity indices, and CWM between non‐invaded, single‐species invaded, and multi‐species invaded local ant communities. We primarily analyzed sample sites where two species, *S. invicta* and *A. gracilipes* , invaded simultaneously, because there was only one sample site where *P. longicornis* and *S. invicta* invaded simultaneously, which did not allow for Mann–Whitney U analysis. Additionally, we utilized permutation multivariate analysis of variance (PERMANOVA) with 9999 permutations to quantify differences in taxonomic and functional composition, turnover, and nestedness among non‐invaded, single‐species invaded, and multi‐species invaded communities.

#### 2.4.4 Software

All analyses were performed in *R*. The *TPD* package (Carmona et al. 2019) used to calculate functional diversity indices and functional similarity. The *FD* package (Van Hengstum et al. 2014) used to calculate CWM. The *betapart* package (Baselga et al. 2018) used for beta diversity analysis. The *lme4* package (Bates et al. 2015) used to construct linear mixed effects models.

## 3 Results

The results of the analyses at the pitfall trap level are as follows:

### 3.1 Impact of Invasive Species Abundance on Native Species Richness and Abundance

Invasion intensity significantly affected native ant communities across different scenarios, as shown in Table 1. During the dry (cool) season, the abundance of invasive species within pitfall traps had no significant effect on native species richness or abundance, except *P. megacephala* (Table 1). During the wet (warm) season, the abundance of *P. megacephala* and *S. invicta* had significant and strong effects on native species richness (−1.83 and −0.29, respectively) and abundance (−0.29 and −0.23, respectively), but no effects were observed in the presence of *A. gracilipes* or *P. longicornis* . Under the co‐invasion scenario, when *A. gracilipes* and *S. invicta* are simultaneously present in the wet season, both native species richness and abundance were negatively impacted (*p* < 0.05). In the *P. longicornis* and *S. invicta* co‐invasion scenario, the native species richness and abundance were not significantly different from non‐invaded areas.

### 3.2 Effects of Invasion Intensity on Taxonomic Diversity

Pitfall traps containing either *S. invicta* and *A. gracilipes* exhibited significant differences in species diversity compared to pitfall traps containing only native species (*p* < 0.001, Table 1). Specifically, the invasion intensity of these two species had a significant negative impact on native α diversity, with values of −0.88 and −0.85, respectively. Traps with *P. megacephala* also exhibited a marked disparity in α diversity compared to non‐invaded traps, while invasion by *P. longicornis* had no significant impact on native diversity. In the co‐invasion scenario of *P. longicornis* and *S. invicta* , α diversity shows a significant positive effect. When the ratio of the invasion intensity of *A. gracilipes* and *S. invicta* is greater than 0.24 and lower than 2.05 under the co‐invasion scenario, the α diversity of native species is higher than in non‐invaded traps. The co‐invasion of *S. invicta* and *P. longicornis* resulted in a notably enhanced positive effect, with the combined impact reaching an effect value of 2.42. When the ratio of the invasion intensity of *S. invicta* and *P. longicornis* is greater than 0.33 and lower than 1.47 (0.33, 1.47) under the co‐invasion scenario, the α diversity of native species is higher than in non‐invaded traps.

Distance‐based redundancy analysis (db‐RDA) was conducted on pitfall traps containing single invasion and non‐invaded traps to evaluate taxonomic differences (see Table 2). In traps containing *P. megacephala* (69 traps), β‐diversity was not calculated due to the complete absence of native species, except for three traps. For pitfall traps with *S. invicta* (*N* = 94), *A. gracilipes* (*N* = 106), and *P. longicornis* (*N* = 73), a pattern of high nestedness and low turnover was observed, with invasive species accounting for 27%, 27%, and 51% of the nestedness, respectively. In contrast, traps with co‐invasions showed lower nestedness values, with 1% for *P. longicornis* and *S. invicta* and 18% for *A. gracilipes* and *S. invicta* .

**TABLE 2:** Results of db‐RDA tests for dissimilarities between uninvaded and invaded (Different invasion situations) communities in their observed taxonomic compositions. Asterisks indicate statistical significance (*** p < 0.001, ** p < 0.01, * p < 0.05). Pitfall traps invaded with *P. megacephala* not shown here (see text for details).

Invaded species	Component	*F*	*R* ^2^	*p*
*S. invicta*	Total dissimilarity	7.46	0.08	< 0.001***
	Turnover	2.72	0.03	< 0.05*
	Nestedness	35.53	0.27	< 0.001***
*A. gracilipes*	Total dissimilarity	7.99	0.07	< 0.001***
	Turnover	0.96	0.009	0.52
	Nestedness	39.4	0.27	< 0.001***
*P. longicornis*	Total dissimilarity	6.93	0.11	< 0.001***
	Turnover	0.48	0.008	0.97
	Nestedness	61.2	0.51	< 0.001***
*A. gracilipes/S. invicta*	Total dissimilarity	1.02	0.03	0.25
	Turnover	0.69	0.002	0.59
	Nestedness	6.56	0.18	< 0.01**
*P. longicornis/S. invicta*	Total dissimilarity	0.03	0.002	0.99
	Turnover	0.05	0.004	0.99
	Nestedness	0.19	0.01	0.86

### 3.3 Effects of Invasion Intensity on Functional Diversity

**FIGURE 1:**
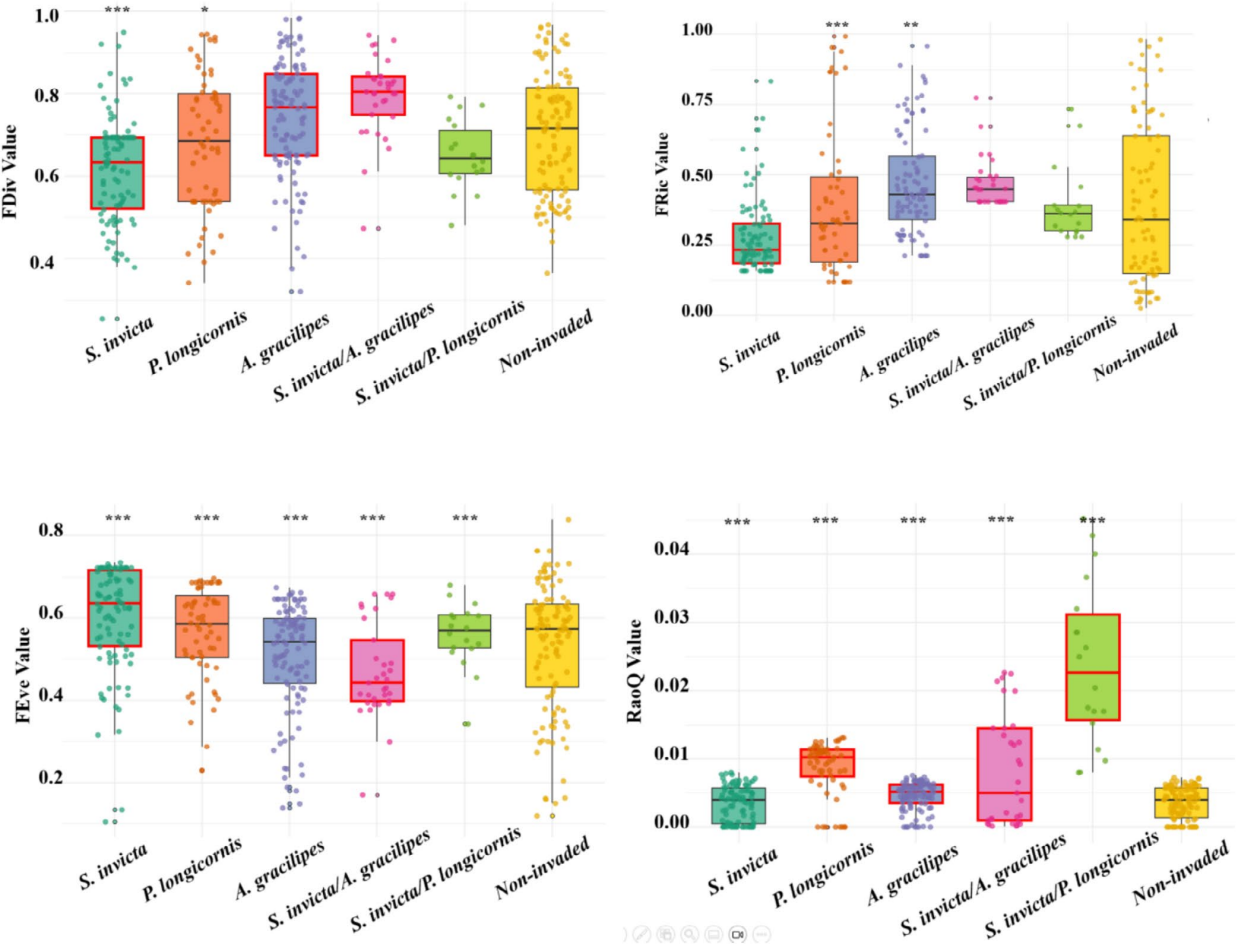
Box plots show the observed values of four functional diversity indices FDiv (Top left), Fric (Top right), FEve (Lower left), and RaoQ (lower right) in pitfall traps. The dots represent the values of individual trap communities, the thick bars represent the mean, the boxes represent the interquartile range, and the vertical lines extend to the maximum and minimum values (excluding outliers). The red boxes represent values that are significantly different from those of native species. The asterisk indicates that the impact of invasion intensity on the value is statistically significant (***p < 0.001, **p < 0.01). For *P. megacephala* invaded pitfall trap communities, considering that more than 95% of the pitfall traps contained only one species, they were not included in the box plot.

Pitfall traps with single invasions by *P. megacephala* showed significant differences for all functional diversity indices when compared to non‐invaded traps. The same was observed for *S. invicta* invasions, except for RaoQ being insignificant. For *A. gracilipes* , we found that FEve and FDic had significant differences compared to non‐invaded pitfall traps. For the co‐invasion scenarios, RaoQ, FEve, and FDiv showed significant differences between *A. gracilipes* and *S. invicta* co‐invasion and non‐invaded traps, while only the RaoQ and Fric exhibited a significant difference between *S. invicta* and *P. longicornis* co‐invasion and non‐invaded traps (Figure 1).

The intensity of invasion by *P. longicornis* , *A. gracilipes* , and *S. invicta* was significantly negatively correlated with FRic, FRed, and RaoQ. However, for *P. megacephala* , functional diversity did not show a significant effect (Table 3). Further analysis revealed that for *S. invicta* and *P. longicornis* co‐invasion scenario, there was a significant positive correlation between the co‐invasion effect and FEve. When the ratio of the invasion intensity of *A. gracilipes* and *S. invicta* is greater than 0.8 and lower than 22 (0.08, 22) under the co‐invasion scenario, FEve and RaoQ were higher than those in the uninvaded traps. Similarly, in the *S. invicta* and *P. longicornis* scenario, RaoQ increased when the ratio ranged from 0.002 to 21.4 (0.002,21.4).

**TABLE 3:** Effects of invasion intensity on functional diversity indices. The “Antagonism” row indicates the range of invasion intensity ratios between co‐invading species under which antagonistic interactions produce a combined impact smaller than the sum of single‐species effects. Values are shown only when both the co‐invasion effect and relevant single invasion effects are statistically significant. “N.A.” denotes conditions where antagonism could not be calculated due to non‐significant effects. The bold text indicates statistically significant differences between invaded and uninvaded pitfall traps. The asterisk indicates that the impact of invasion intensity on the value is statistically significant (***p < 0.001, **p < 0.01).

Invasion	FRic	FEve	FDiv	FRed	RaoQ
*S. invicta*	**−0.48**	0.36***	−0.40***	−0.46*	−0.01***
*A. gracilipes*	−2.15**	0.49***	**−0.06**	−1.25***	−0.01***
*P. longicornis*	−1.39***	0.45***	−0.35*	−0.72**	−0.02***
*P. megacephala*	**−0.79**	**1.49**	**1.37**	**−7.02**	**0.04**
*S. invicta/A. gracilipes*	−1.27	4.60***	**−0.24**	−2.23	0.24***
Antagonism	N.A.	N.A.	N.A.	N.A.	(0.08,22)
*S. invicta/P. longicornis*	−0.11	4.59***	1.35	0.77	0.43***
Antagonism	N.A.	N.A.	N.A.	N.A.	(0.002,21.4)

**FIGURE 2:**
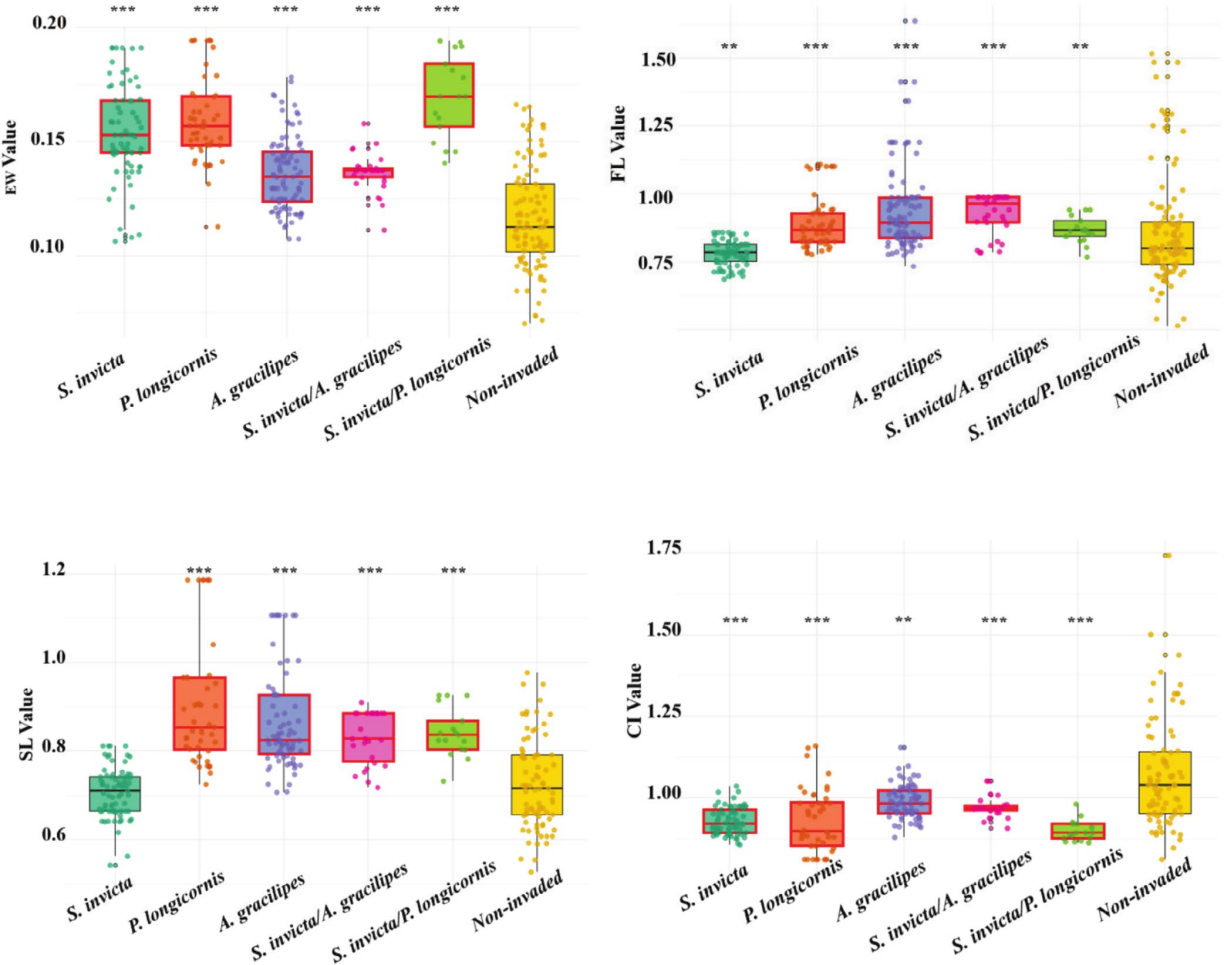
Box plots show the observed values of four CWM indices for size‐corrected EW (Top left), FL (Top right), SL (Lower left), and CI (lower right) in pitfall traps. The dots represent the values of individual trap communities, the thick bars represent the mean, the boxes represent the interquartile range, and the vertical lines extend to the maximum and minimum values (excluding outliers). The red boxes represent values that are significantly different from those of native species. The asterisk indicates that the impact of invasion intensity on the value is statistically significant (***p < 0.001, **p < 0.01). For *P. megacephala* invaded pitfall trap communities, considering that more than 95% of the pitfall traps contained only one species, they were not included in the box plot.

CWM analysis revealed that further calculations could not be conducted for *P. megacephala* due to the low number of pitfall traps containing native species (*N* = 3 out of 69). For the other three species, significant differences were observed in EW, CI, and Prw between invaded and non‐invaded traps. In traps with co‐invasion, significant differences were found in EW, SL, and CI when compared to non‐invaded communities (Figure 2). Invasion intensity analysis showed that *S. invicta* was significantly positively correlated with CI. Under co‐invasion scenarios, *S. invicta* and *A. gracilipes* exhibited a significant decreasing trend in CI with increasing co‐invasion effect.

### 3.4 Site Level

Sites with single invasions by *A. gracilipes, S. invicta, P. longicornis*, and *P. megacephala* showed significant differences in α diversity compared to non‐invaded sites (*p* < 0.05). No significant differences in α diversity were observed in cases of co‐invasions (*p* > 0.05). For β diversity, only *A. gracilipes* single invasions exhibited significant nestedness, accounting for 88% of β diversity. The sites invaded by one of the four invasive species showed significant differences from non‐invaded sites in terms of RaoQ and FRic (*p* < 0.05). Additionally, *A. gracilipes* invasion significantly affected FDiv (*p* < 0.05), while co‐invasions of *A. gracilipes* and *S. invicta* showed significant changes in FRic (*p* < 0.05). Functional β diversity analysis indicated significant functional turnover in sites invaded by *A. gracilipes* (77%) and *P. longicornis* (72%). Functional β diversity for *P. megacephala* could not be calculated due to the quasi absence of native species.

## 4 Discussion

This study explores the impacts and mechanisms by which several invasive species, their invasion intensity, and co‐occurrences affect local biodiversity. Our results indicate that invasive species effects on invaded communities appear to be heterogeneous among and between species in function of the invasion intensity, invasive species identity, and co‐invasion scenario. The increase in invasion intensity of these species leads to a systematic decline in local species diversity. The invasion of a single species causes selective replacement, resulting in the systematic loss of sensitive species. Further analysis of functional diversity reveals that different invasive species exert differential regulation on functional diversity indices (such as RaoQ and FDiv) and community‐weighted means (CWM). Interestingly, the coexistence of multiple invasive species was associated with less pronounced changes in both functional diversity and taxonomic composition compared to single‐species invasion scenarios. This comparatively limited divergence from uninvaded communities may indicate that antagonistic interactions among co‐invaders dampen their individual ecological impacts. Such dynamics could contribute to a temporary stabilization or partial recovery of local biodiversity under specific conditions such as early‐stage co‐invasion.

### 4.1 Impacts of Single Invasions

Numerous studies indicate that invasive species lead to negative ecological changes for native species and represent one of the main drivers of community diversity changes among relatively undisturbed global systems of animals (Holway and Suarez 2006; Moran and Alexander 2014; Tercel et al. 2023). The ecological impacts of invasive species are often density‐dependent, with results from a meta‐analysis finding that the increased abundance of invasive species is usually associated with an increased decline in native species richness (Bradley et al. 2019). Our results concur; during the wet season, increased abundance of *S. invicta* and *P. megacephala* leads to differential impacts on native species. At the pitfall trap level, *P. megacephala* abundance exhibits a stronger negative correlation with native species richness and abundance than *S. invicta* . This suggests that in the region studied, invasions of *P. megacephala* generate the strongest native species loss. Indeed, in half of the sites studied, native species were entirely absent. This may be attributed to its pronounced territorial behavior and aggressive interactions with other ant species, which can result in significant reductions in native species diversity (Hoffmann et al. 1999; Wetterer 2007). In contrast, in sites dominated by *S. invicta* , a number of native species survived, as shown by the values of α diversity, despite the invasion.

Although taxonomic β diversity shows that the invasion processes of all single invasive species significantly alter the community structure (nestedness), not all invasive species have a significant effect on local taxonomic α diversity. Some species' invasions have relatively limited impacts on local taxonomic diversity. For example, the abundances of *P. longicornis* and *A. gracilipes* show mixed impacts on local communities (−1.12 and −0.85 respectively, Table 1). For sites invaded by *P. longicornis* and *A. gracilipes* (at the site level), the local communities exhibited significantly different α diversity compared to non‐invaded sites, indicating that both invasive species altered local species diversity patterns. Concurrently, declines in RaoQ and FRic at this scale suggest that invasive species may reduce functional trait differences in the original community, leading to a convergence in ecological functions. This could potentially lead to a decrease in the stability and resilience of the local communities (Jiang et al. 2015; Galland et al. 2020).

At the smallest scale (pitfall trap level), functional diversity analysis reveals that communities invaded by *A. gracilipes, P. longicornis*, or *S. invicta* show significant signals of functional convergence to varying degrees. This variation stems from differences in the ecological dominance and behavioral traits of each invasive species, as well as the sensitivity of individual functional diversity metrics. For instance, *A. gracilipes* produces the most pronounced decline in FRic (−2.15) and FRed (−1.25), suggesting strong displacement of functionally unique species. In contrast, *P. longicornis* exerts moderate reductions in FRic (−1.39) and FRed (−0.72), possibly due to the lower aggressiveness of this species (Bertelsmeier et al. 2015). *Solenopsis invicta* , known for its competitive exclusion of other foragers, induces significant drops in FDiv (−0.40) and RaoQ (−0.01), indicative of trait clustering and reduced functional dispersion (Zhou et al. 2014). Changes in these functional diversity indices indicate that the invasion by a single species usually compresses the range of functional traits in the invaded community and concentrates traits in a specific direction, resulting in a reduced and homogenized functional diversity. This pattern is consistent with documented ant invasion cases in Hong Kong, although in a different region, where the invasion of *S. invicta* has been shown to induce functional clustering in local communities by competitively excluding unique foragers (Wong et al. 2020). Similar results have also been observed in other flora and fauna, such as in alien plants invading the northern Adriatic Sea region and leading to functional homogenization of coastal ecosystems (Tordoni et al. 2019), indicating the widespread nature of this phenomenon. Moreover, our results show that with increasing invasion intensity, FEve rises, although this increase may be superficial. Overall, most ant invasions studied here lead to a reduction in FRic, forcing the remaining species to occupy narrower niche spaces. This spatial compression results in a “pseudo‐evenness” pattern where functional traits are distributed more evenly within a diminished trait space not because of true ecological balance, but due to the loss of functionally distinct species and convergence around a limited set of traits. In this scenario, the increase in FEve does not necessarily reflect a healthy or diverse community structure, but rather a homogenized configuration shaped by trait filtering and ecological displacement.

A significant increase in functional redundancy (FRed) with rising invasion intensity of all single invasions suggests substantial structural and functional shifts within the community. This systemic reduction in FRed can have compound effects: the convergence of specific functional traits (such as head morphology or specialized locomotory organs) which diminishes the functional diversity dimension, while the community's reduced ability to buffer species loss through functional FRed may exacerbate the degradation of ecosystem services provided (Bihn et al. 2010; Nooten et al. 2022). In addition, the functional traits related to body size did not exhibit significant changes within communities invaded by *S. invicta* . This lack of variation could be attributed to the polymorphic nature of the invasive species, which includes a broad range of body sizes that may mask the impact of the invasion on this particular trait dimension.

The RaoQ indicates a significant downward trend with increasing invasion intensity, suggesting a systematic decline in community functional diversity post‐invasion. This observation aligns closely with findings in Argentine ant ( *Linepithema humile* ) invaded areas, where ant community recovery rates lag behind those of naturally recovering areas (Frasconi Wendt et al. 2021). The CWM shows that in *S. invicta* ‐invaded pitfall trap, the cephalic index (CI) increases significantly with invasion intensity and displays significant differences from native species at both invasion intensities. This indicates that under invasion pressure, native species with lower CI may be suppressed by *S. invicta* , leading to an increase in the community CI. Larger CI is typically associated with stronger mandible muscles (Kikuchi et al. 2008), potentially adapting to predation and cutting hard food items (such as seeds or insect remains). The increase in CI may also imply that native ants are forced to shift from a “rapid resource discovery” strategy to an “enhanced combat ability” strategy to cope with resource monopolization by red imported fire ants. For *A. gracilipes* and *P. longicornis* ‐invaded pitfall traps, the invasions lead to increased SL and FL, reflecting a community tendency towards enhancing mobility and resource‐tracking capabilities as a survival strategy or indicating that species with high mobility and resource‐tracking efficiency become dominant.

The functional diversity of *P. megacephala* invasion (pitfall trap level) reveals that although significant functional trait convergence was not detected in functional diversity indices (such as RaoQ), FDiv shows a significant positive correlation with the invasion intensity gradient, and deeper ecological disturbances are unveiled based on community data. From 69 pitfall traps with this invasive species, native species were completely absent in 66 (or 95.6%). This scenario suggests that *P. megacephala* may significantly compress the ecological niche space of native species through competitive exclusion (or predation). The study was conducted in habitats near small rural villages located at the edge of dense forests, relatively unaffected in recent decades by urban development or heavy anthropogenic disturbances. This setting helps isolate the ecological impact of *P. megacephala* without the confounding influence of significant anthropogenic disturbances. Although current functional diversity indices may not fully capture the intensity of its ecological impact, the extreme scarcity of native species (< 5%) provides a strong indication that *P. megacephala* invasion may exert the most severe consequences on the local communities.

### 4.2 Impact of Co‐Invasions

In the co‐invasion scenario of *P. longicornis* and *S. invicta* , no significant correlation between the individual abundance of invasive species and the species richness and abundance of native species was detected. When *P. longicornis* co‐invades with *S. invicta* , the antagonistic effect reduces the impact of co‐invasion on local α diversity, compared to the single impact of *S. invicta* (invasion intensity ratio of the two species must be in the range of 0.33 to 1.47). In the co‐invasion of *A. gracilipes* and *S. invicta* , the antagonistic effect is similarly pronounced, leading to approximately a 2.66 reduction in α diversity for each unit increase in invasion intensity. In this case, the antagonistic effect between the two species may outweigh the cumulative effect of their respective single invasions as well. In other words, when two species invade together (Occur simultaneously in the same pitfall trap), their negative impact on native α diversity may be lower than when they invade individually, or even, in some cases, positively affect α diversity (invasion intensity ratio of the two species must be in the range of 0.24 to 2.05).

This phenomenon can be attributed to the complex interactions between two invasive species in terms of competition for resources, ecological niche differentiation, and mutual constraints. In co‐invasion scenarios, the two species may compete with or suppress each other, thereby weakening the influences on native species and leading to a reduced impact on native α‐diversity (Jackson 2015; Ahmad et al. 2025). Furthermore, taxonomic β diversity analysis shows that in co‐invasion systems, the impact of nestedness was reduced in comparison to single invasion scenarios. This suggests that the species loss gradient caused by co‐invasion is less severe than that caused by a single invasion. Similar findings have been reported in plant communities, with the antagonistic interactions between *Solidago canadensis* and *Juglans regia* during co‐invasion presenting a smaller impact on native species diversity compared to single‐species invasions Lenda et al. (2019). This is further supported through meta‐analysis on co‐invasions, showing that the interactions among invasive species can moderate their impacts on ecosystems, resulting in lower overall impacts from co‐invasions than from individual invasions (Jackson 2015).

At the site level, the functional diversity of communities invaded by multiple invasive species ( *A. gracilipes* and *S. invicta* ) shows a significant decline in FRic, indicating that co‐invasions lead to a reduction in the range of functional traits within the community, thereby shrinking the overall “functional space” occupied by the community. However, CWM values reveal that co‐invasions do not significantly affect the community trait indices. This suggests that invasive species may compress the community's functional space through functional replacement (although the components of functional β diversity [replacement and turnover] did not show significant differences between invaded and uninvaded sites), while maintaining core functional traits in the short term.

At a smaller scale (pitfall trap level), functional diversity reveals similar characteristics for different types of co‐invasions. For the co‐invasion of *A. gracilipes* and *S. invicta* , as invasion intensity increases, the antagonistic effect of RaoQ shows an increasing trend (0.24). The results show that when the invasion intensity ratio of two invasive species is in the range of 0.08 to 222, the impact of antagonism on some functional diversity will exceed the cumulative effect of the two single invasive species. Compared with single invasions (FRed and RaoQ all decreased significantly), the co‐invasion of *A. gracilipes* and *S. invicta* ants may lead to mutual inhibition, resulting in a reduction in the ecological competitive advantage of both sides, alleviating the competitive exclusion pressure caused by a single dominant invasive species (Ahmad et al. 2025).

In the case of co‐invasion by *P. longicornis* and *S. invicta* , the functional trait differences are significantly increased within the invaded communities (increased in RaoQ). Under certain conditions (ratio range from 0.002 to 21.4), the antagonistic effect of the co‐invasion may result in some functional diversity indicators (e.g., RaoQ) becoming higher than in the uninvaded sample sites. Combining the co‐invasion by *P. longicornis* and *S. invicta* with that by *A. gracilipes* and *S. invicta*, both scenarios lead to an increase in community taxonomic α diversity and a decrease in nestedness of taxonomic β diversity. These results all indicate that, under certain circumstances, co‐invasion may, to a certain extent, enhance the diversity and stability of community functions and is more conducive to maintaining ecosystem balance than single species invasion.

The results demonstrate that at specific invasion intensity ratios (e.g., 0.08–22 or 0.002–21.4), antagonistic interactions between co‐invasive species can alleviate the negative impacts of invasion on the α‐diversity of native communities. These antagonistic interactions could thus preserve certain ecological functions through functional substitution. However, the realization of this “balance” is subject to various limitations and constraints. First, the ratio of invasion intensity between invasive species meets a specific requirement, which is dependent on natural conditions that are difficult to regulate artificially. Secondly, in the case of co‐invasions, the RaoQ index exhibits antagonistic effects rather than a consistent decline, indicating that co‐invasion dynamics can generate functional differentiation rather than strictly driving homogenization. This shift suggests that under certain conditions, competition between invasive species may result in functional divergence, mitigating the immediate loss of ecological resilience. However, despite these short‐term buffering effects, the long‐term ecological consequences remain uncertain. While functional redundancy may help sustain key ecosystem processes, prolonged antagonistic interactions could destabilize trait distributions, influencing species interactions in unpredictable ways. More critically, this dynamic balance of invasion antagonism is unsustainable and changes with natural environment dynamics (e.g., climate change, anthropogenic disturbances) that could easily break this fragile window of equilibrium, transforming invasive species antagonisms into synergistic effects and exacerbating biodiversity loss. Therefore, although research has revealed the potential ecological value of mutual constraints (antagonism) among invasive species, in practical management, prevention of new invasions and control of existing invasive species should be prioritized as a strategy to avoid placing ecosystem survival on high‐risk dynamic equilibrium.

## 5 Limitations and Future Directions

Although our analysis reveals significant patterns regarding the impacts of invasive species in both single invasions and co‐invasions, it is important to acknowledge that the sample size, particularly for co‐invasion scenarios, is relatively limited. The restricted number of sites in these complex contexts may constrain the statistical robustness of the observed trends, potentially emphasizing effects that might differ with broader sampling. Indeed, alternative or more varied sampling methods might also reveal additional patterns, particularly under different environmental conditions. For instance, while our findings suggest that antagonistic interactions in co‐invasion cases can partially ameliorate negative impacts on native community diversity, expanding the dataset across additional sites and employing diverse sampling strategies could refine or even modify these observed dynamics. Consequently, while the current results provide valuable preliminary insights, further investigations with larger, more representative samples and varied sampling approaches are essential to confirm the consistency and generality of these findings. Future research should not only integrate broader spatial and temporal scales but also incorporate a systematic focus on co‐invasion scenarios, thereby enhancing our understanding of the complex interplay among invasive species. This expanded perspective is crucial not only for reinforcing the robustness of our study but also for guiding effective management strategies in ecosystems increasingly challenged by the simultaneous presence of multiple invaders.

## Author Contributions

**Jiaxin Hu:** conceptualization (equal), data curation (lead), investigation (equal), methodology (equal), visualization (lead), writing – original draft (lead), writing – review and editing (equal). **Taylor A. Bogar:** investigation (equal), writing – review and editing (equal). **Matthew T. Hamer:** investigation (equal), writing – review and editing (equal). **Benoit Guénard:** conceptualization (equal), methodology (equal), funding acquisition (lead), supervision (lead), writing – review and editing (equal).

## Conflicts of Interest

The authors declare no conflicts of interest.

## Supporting information


**Data S1:** ece372095‐sup‐0001‐Supinfo01.docx.

## Data Availability

The original contributions used in all analyses for this study are available at Supporting Information and https://connecthkuhk‐my.sharepoint.com/:f:/g/personal/u3008597_connect_hku_hk/EiG28t0aQhdJkQ7P1Fv4‐YMB‐UIGIc5eVc1slSOrJ9I_kQ?e=6scgHh.
