# Supplementary material for: Impacts of Single and Multiple Co‐Existing Invasive Species on Subtropical Native Ant Communities
Source: Ecol Evol. 2025 Sep 1;15(9):e72095. doi: 10.1002/ece3.72095 (PMC12401564; doi:10.1002/ece3.72095)
Supplement: Supplementary file 1 — Data S1: ece372095‐sup‐0001‐Supinfo01.docx. [file ECE3-15-e72095-s001.docx]

Table S1: Differences in species and functional diversity indices between pitfall traps with invasive species and those without in the invasion sample sites. Asterisks denote statistical significance (*** p < 0.001; ** p < 0.01).

|  | *Solenopsis invicta* | *Anoplolepis gracilipes* | *Paratrechina longicornis* | *Pheidole megacephala* |
| --- | --- | --- | --- | --- |
| α diversity | 293.5*** | 1454** | 1850*** | 140*** |
| FRic | 670 | 760*** | 872** | 620*** |
| FEve | 338*** | 1293 | 1521 | 2*** |
| FDiv | 935 | 900** | 1205 | 554*** |
| FRed | 1231*** | 1066 | 1180 | 478*** |
| RAOQ | 1491*** | 1356 | 815*** | 654*** |

Table S2: The seven traits measured for each individual, and hypothesized correlations between these traits and the overall performance and fitness of ants.

| Trait | Measurements | Hypothesized link to performance and fitness |
| --- | --- | --- |
| Body size | Weber's length: diagonal length of mesosoma | Pertains to mass-dependent metabolic demands and also impacts the utilization and acquisition of resources (Silva & Brandão 2010). |
| Mandible length | Length of left mandible | Reflects the selective pressures associated with dietary type and specialization (Silva & Brandão 2010). |
| Scape length | Length of scape of left antenna | Responds to selection on navigation and sensory abilities (Silva & Brandão 2010). |
| Pronotum width | Width of pronotum | Determines the extent of spatial accessibility for resource acquisition and the volume of musculature necessary for head support and load-bearing capacities (Keller, Peeters & Beldade 2014; Schofield, Bishop & Parr 2016) |
| Cephalic index | Head width /Head Length | Related to jaw muscles used for foraging, may influence aggression and handling time (Kikuchi *et al.* 2008). |
| Eye width | Width of left eye | dictates the capacity for navigation, foraging efficiency, and the detection of both predators and prey, while also serving as an indicator of active periods (Silva & Brandão 2010) |
| Femur length | Length of left hind femur | Associated with search area and  foraging speed (Pearce-Duvet, Elemans & Feener Jr 2011). |

Table S3: The table summarizes the ant sample data information collected at each study location (Location 1 to 4). Locations 1~4 represent the coordinates of the sample area. The "sampling season" column records in detail the time and season information when the sampling occurs, where "22", "23" and "24" represent the sampling activities in 2022, 2023 and 2024 respectively; marks such as "sum" and "win" indicate the sampling periods in summer and winter respectively. If the "sampling season" in a record contains multiple identifiers (for example, "22sum, 22win, 23sum"), it means that sampling was carried out in the corresponding seasons in the corresponding years.

| species | *S. invicta* | *A. gracilipes* | *P. longicornis* | *P. megacephala* | *S. invicta/ A. gracilipes* | *S. invicta/ P. longicornis* | Non invaded |
| --- | --- | --- | --- | --- | --- | --- | --- |
| Location 1 | 22.319401, 114.05163 | 22.29634, 113.96241 | 22.29309, 113.95484 | 22.46643, 114.18063 | 22.29750, 113.96768 | 22.31833, 114.04526 | 22.40355, 114.10879 |
| sampling season | 22sum, 22win, 23sum | 22sum, 22win, 23sum, 23win | 22sum, 22win, 23sum | 22sum, 22win, 23sum | 22sum, 22win, 23sum, 23win | 22sum, 22win, 23sum | 22sum, 22win, 23sum |
| Location 2 | 22.448037, 114.086034 | 22.49000,114.18329 | 22.26550, 114.135565 | 22.2882，113.9197 | 22.297175, 113.96513 |  | 22.40465, 114.107696 |
| sampling season | 23sum, 23win | 23sum, 23win | 23sum, 23win | 23win, 24sum | 22sum, 22win, 23sum, 23win |  | 23sum, 23win |
| Location 3 | 22.4495,114.0851 | 22.49027,114.18243 | 22.26553, 114.13430 | 22.2878，113.9190 | 22.31875, 114.05021 |  | 22.40421, 114.108219 |
| sampling season | 23sum, 23win | 23sum, 23win | 23sum, 23win | 23win, 24sum | 23win, 24sum |  | 23sum, 23win |
| Location 4 | 22.31850, 114.04355 | 22.297113, 113.96412 | 22.26634, 114.13538 | 22.2867，113.9186 |  |  | 22.40288, 114.10836 |
| sampling season | 23sum, 23win | 23sum, 23win | 23sum, 23win | 23win, 24sum |  |  | 23sum, 23win |

Table S4: The table introduces the functional diversity indicators commonly used in ecological research.”

| Functional Index | Full name | Definitions |
| --- | --- | --- |
| FRic | Functional Richness | volume of multidimensional trait space occupied |
| FEve | Functional Evenness | evenness of trait distribution within occupied space |
| FDiv | Functional Divergence | the degree of dispersion of functional traits within the community |
| FRed | Functional Redundancy | the extent to which trait values are represented by multiple species within the community |
| RaoQ | Rao’s Quadratic Entropy | comprehensively considers species richness and functional trait differences. |

References:

Keller, R.A., Peeters, C. & Beldade, P. (2014) Evolution of thorax architecture in ant castes highlights trade-off between flight and ground behaviors. *Elife,* **3,** e01539.

Kikuchi, T., Miyazaki, S., Ohnishi, H., Takahashi, J., Nakajima, Y. & Tsuji, K. (2008) Small queens and big-headed workers in a monomorphic ponerine ant. *Naturwissenschaften,* **95,** 963-968.

Pearce-Duvet, J.M., Elemans, C.P. & Feener Jr, D.H. (2011) Walking the line: search behavior and foraging success in ant species. *Behavioral Ecology,* **22,** 501-509.

Schofield, S.F., Bishop, T.R. & Parr, C.L. (2016) Morphological characteristics of ant assemblages (Hymenoptera: Formicidae) differ among contrasting biomes. *Myrmecological News,* **23,** 129-137.

Silva, R.R. & Brandão, C.R.F. (2010) Morphological patterns and community organization in leaf‐litter ant assemblages. *Ecological Monographs,* **80,** 107-124.
